# A designed peptide targeting CXCR4 displays anti-acute myelocytic leukemia activity in vitro and in vivo

**DOI:** 10.1038/srep06610

**Published:** 2014-10-14

**Authors:** Xiaojin Li, Hua Guo, Yanlian Yang, Jie Meng, Jian Liu, Chen Wang, Haiyan Xu

**Affiliations:** 1 Institute of Basic Medical Sciences, Chinese Academy of Medical Sciences & Peking Union Medical College, Beijing 100005, P. R. China; 2 National Center for Nanoscience and Technology, Beijing 100190, P. R. China

## Abstract

Leukemia cells highly expressing chemokine receptor CXCR4 can actively response to stroma derived factor 1α (CXCL12), trafficking and homing to the marrow microenvironment, which causes poor prognosis and relapse. Here we demonstrate that a novel designed peptide (E5) targeting CXCR4 inhibits CXCL12- and stroma-induced activation in multiple acute myelocytic leukemia (AML) cell lines and displays anti-AML activity. We show that E5 has high affinity to multiple AML cells with high CXCR4 level in a concentration dependent manner. E5 significantly inhibits CXCL12- or murine stromal cell (MS-5)-induced migration of leukemia cells and prevents the cells from adhering to stromal cells. Mechanistic studies demonstrate that E5 down-regulates CXCL12-induced phosphorylation of Akt, Erk, and p38, which affects the cytoskeleton F-actin organization and ultimately results in the inhibition of CXCL12- and stroma-mediated leukemia cell responses. E5 can induce concentration-dependent apoptosis in the four AML cell lines tested while did not affect the viability of MS-5 or human umbilical vein cell (ea.hy926) even at 80 µM, both of which have a low level of CXCR4. *In vivo* experimental results show that immunocompromised mice transplanted with HL-60 cells survived longer when treated with E5 twice a week in comparison to those treated with cyclophosphamide.

Critical roles of the tumor microenvironment in conferring drug resistance as a major cause of relapse and incurability of tumors have been widely recognized with evidences being accumulated^1^,^2^,^3^,^4^. For example, stromal cells in the bone marrow protect acute myeloid leukemia (AML) and chronic lymphocytic leukemia from the apoptosis induced by chemotherapy^5^. The stroma-secreted chemokine, stroma derived factor 1α (SDF-1α, or termed as CXCL12), and its cognate receptor, the chemokine receptor 4 (CXCR4) are two very key mediators in the cross-talking between tumor cells and their microenvironment^6^. The interaction between the two mediators (also termed as CXCR4/CXCL12 axis) can induce various cellular behaviors, such as migration, adhesion, invasion and infiltration^7^,^8^. Many leukemia cells have elevated CXCR4 levels^9^, which allows them not only to acquire high ability of migration and invasion to targeted organs to cause tumor metastasis but also to home to the bone marrow to get favorable conditions for survival and growth in responses to CXCL12 secreted by stromal cells or endothelial cells in the microenvironment. It has been reported that acute myelocytic leukemia (AML)^10^, chronic^11^ or acute^12^ lymphocytic leukemia (CLL or ALL) cells utilize the interaction of CXCR4/CXCL12 for homing to marrow stromal cells *in vitro* and *in vivo*. Leukemia cells that adhere to stromal cells through CXCR4 are therefore protected from the effects of cytotoxic chemotherapy and represent a reservoir for minimal residual disease and relapses^1^,^13^.

In view of the great importance of CXCR4/CXCL12 axis in the leukemia development, interfering with stromal/leukemic cell interaction and eradicating leukemia cells through inhibition of CXCR4 by antagonists has become one attractive strategy in the past decades^14^,^15^. Multiple antagonists targeting CXCR4 have been developed for uses in leukemia treatments, which can be divided into three categories: (1) chemical molecules, representatives are AMD3100 and its derivatives AMD3465 and AMD11070, they are originally developed for HIV treatment^16^,^17^,^18^,^19^,^20^,^21^,^22^; (2) peptides obtained from horseshoe crabs^15^,^16^,^17^,^18^, representatives are T140 and its derivatives TC14012 and TN14003, and RCP168 derived from viral macrophage inflammatory protein II^23^; (3) antibodies such as 12G5 and BMS-936564/MDX-1338^11^,^24^. Both chemical molecules and peptides have shown promising potentials of inhibiting leukemia cells from migrating and adhering to the bone marrow microenvironment. It is noticeable that AMD3100 and its derivatives are investigated not only in lymphocytic but in myelocytic leukemia therapies^25^, however peptides are mainly reported in lymphatic leukemia treatments (ALL and CLL)^16^,^17^,^18^, rarely in myelocytic leukemia yet, and most of them are still in the stage of laboratory research. Therefore, it is very necessary and of great significance to develop novel peptides targeting CXCR4 for providing more therapeutic options in leukemia treatments.

Herein we report a novel peptide (named as E5) by cell-based selection from the *de novo* designed peptides and investigated its inhibitory effect on CXCR4/CXCL12 axis in multiple human acute myelocytic leukemia cell lines, including acute promyelocytic leukemia cell of HL-60 and NB4, acute monocytic leukemia cell of THP-1 and myelomonocytic leukemia cell of U937. We show that E5 inhibits CXCL12-induced and stromal cell MS-5-induced cell migration and adhesion by down-regulating the activation of Erk, Akt and p38/MAPK signaling pathway and affecting actin cytoskeleton reorganization. Furthermore, subcutaneous injection of E5 prolongs the survival of leukemia mice transplanted with HL-60 cells in comparison to the intraperitoneal injection of cyclophosphamide.

## Results

### E5 has high affinity to multiple human acute myelocytic leukemia cells expressing CXCR4

**Figure 1:**
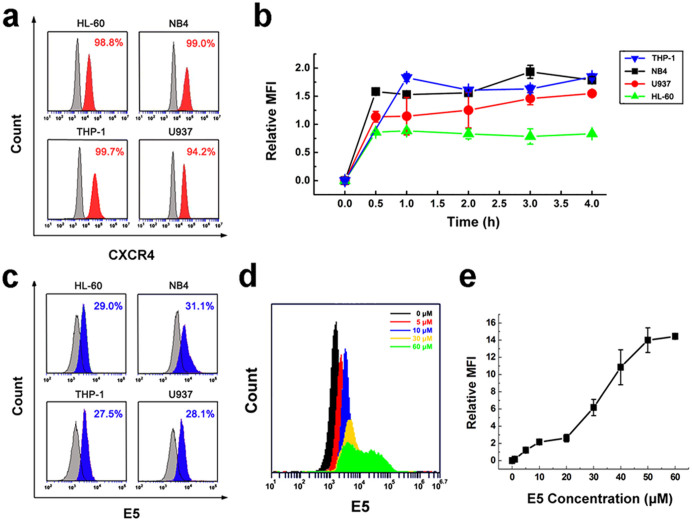
Affinity and kinetic binding of E5 to multiple leukemia cell lines. (a) The CXCR4 level in HL-60, NB4, THP-1 and U937 leukemia cells analyzed by flow cytometry using antibody for CXCR4. (b) Time course of E5 binding amount in the different cells at 10 µM. (c) Affinity of E5 to the different cells at 4 h at 10 µM. Results were obtained from flow cytometry analysis using biotin-labeled E5 and streptavidin-conjugated FITC. (d) and (e) Binding amount of E5 to HL-60 cells after 2 h incubation. The data are presented as mean ± SD (n = 3).

In the first set of experiments, we aimed to determine the affinity of E5 to leukemia cells. Four cell lines including HL-60, NB4, THP-1 and U937 were chosen as cell models because the four cell lines are typical human acute myelocytic leukemia cell lines and highly express CXCR4 in the surface. The CXCR4 level for HL-60, NB4, THP-1 and U937 was detected 98.8%, 99.0%, 99.7% and 94.2% respectively (Fig. 1a). As shown in Figure 1b, the initial binding rate of E5 is associated with the incubation time. The fastest increase in the mean fluorescent intensity (MFI) was observed on NB4, the next two on THP-1 and U937, and the last one on HL-60. The binding platform was reached after 0.5 h incubation and maintained to 4 h in the four cell lines. At the platform stage, the binding amount of E5 to NB4 and THP-1 was similar, while the amount binding to U937 and HL-60 was lower. After 4 h incubation, the percentage of fluorescent cells was 29%, 31.1%, 27.5% and 28.05% for HL-60, NB4, THP-1 and U937 respectively (Fig. 1c). The binding amount of E5 is also concentration dependent (Fig. 1d). As shown in Figure 1e, the MFI of the cells went up gradually when the concentration of E5 was increased from 1 µM to 60 µM, exhibiting a S-shape tendency for three phases, slower increase within 1 µM to 20 µM, faster from 20 µM to 50 µM, and nearly constant from 50 µM to 60 µM.

### E5 has different cytotoxicity to acute myelocytic leukemia cells and nonmalignant cells

**Figure 2:**
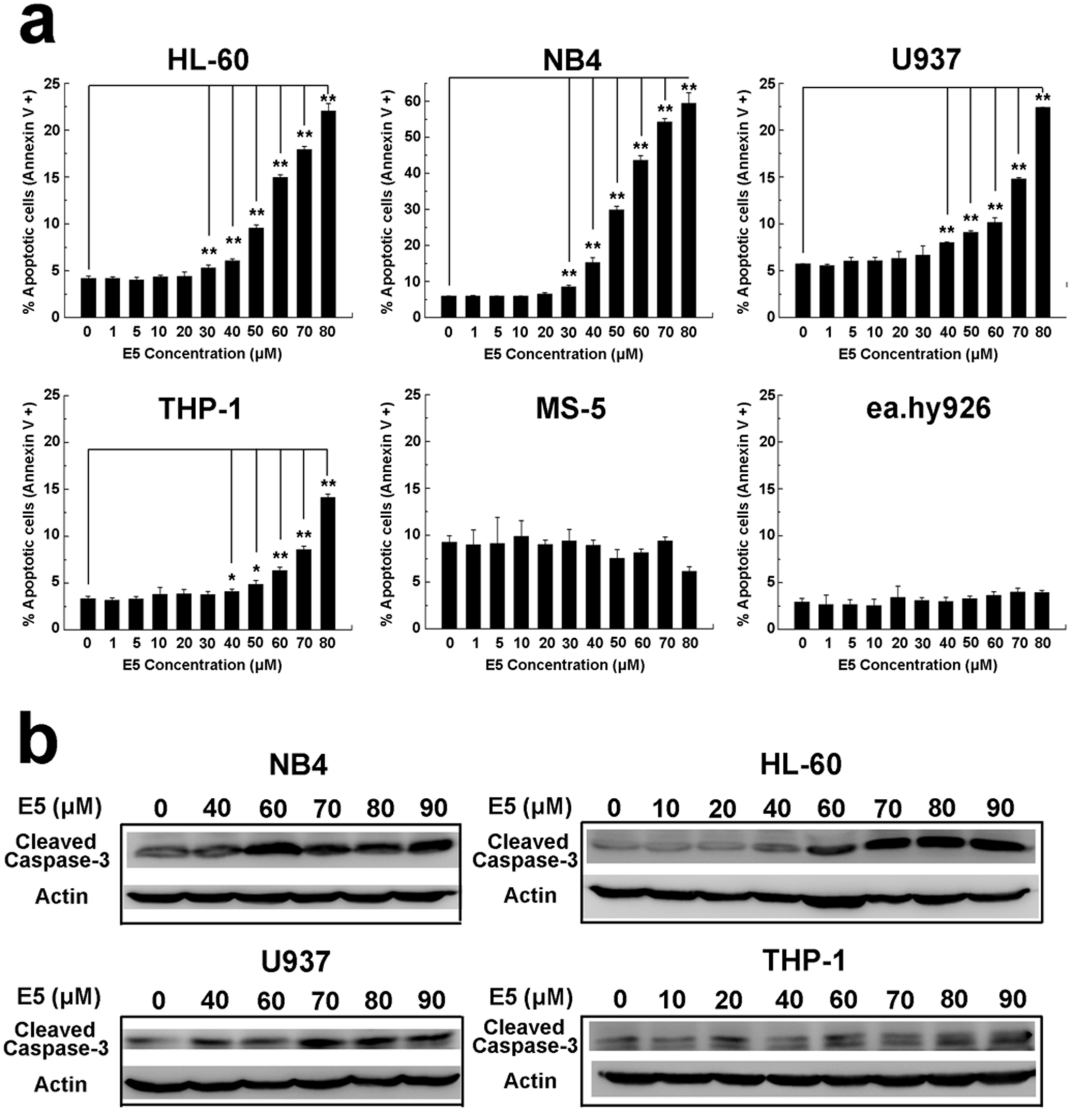
The cytotoxicity of E5 on different cells. (a) Data obtained from the FITC-Annexin V/PI double staining and flow cytometry assay show apoptosis levels for HL-60, NB4, U937, THP1, MS-5 and ea.hy926 cells incubated with E5 at different concentration. The data are presented as mean ± SD (n = 3). The * represents significant difference from sample groups to the control group (*: *p* < 0.05, **: *p* < 0.01). (b) E5 induces the activation of caspase-3 signaling in concentration-dependent manner in HL-60, NB4, U937 and THP-1. Full-length blots are presented in Supplementary Figures.

Next we evaluated the cytotoxicity of E5 using the apoptosis assay by flow cytometry. The four leukemia cell lines as well as two nonmalignant cell lines (murine stromal cell MS-5 and human umbilical vein cell ea.hy926) were investigated upon the exposure of E5. Results from FITC-Annexin V/PI double staining (Fig. 2a) show that among the four cell lines, NB4 is the most sensitive one to E5. The apoptosis percentage of NB4 was reached 16% when E5 concentration was 40 µM, and then reached 30% when E5 was 50 µM, further reached 60% when E5 was 80 µM. For HL-60 and U937, the apoptotic effect of E5 was in a similar level. When E5 concentration was reached 50 µM, the apoptosis percentage was about 10%, and when E5 was increased 80 µM, the apoptosis percentage was 22%. It is seemed that THP-1 is not sensitive enough to E5. The apoptosis percentage was gradually elevated under 9% when E5 was 70 µM, and further reached 14% when E5 was increased to 80 µM. Examinations of caspase-3 signaling indicated that E5 induced the activation apoptotic signaling in a concentration dependent manner in the four cell lines (Fig. 2b), which is consistent with the results of flow cytometry. It is noticeable that E5 did not affect the viability of MS-5 or ea.hy926, both of which have a low level of CXCR4. The apoptosis level for MS-5 or ea.hy926 induced by E5 remained constant and lower than 10% or 5% respectively even when E5 was increased to 80 µM (Fig. 2a). These data suggest that E5 has toxic effect on acute myelocytic leukemia cells, but the effect degree is associated with the cell type. Meanwhile, E5 has a favorable safety to both nonmalignant cells. According to above results, E5 concentration of 10 µM was chosen for the following assay of migration, adhesion, cytoskeleton rearrangement and cellular signaling, because in these assays the viability of cells should be remained when cellular signaling and behaviors are changed by E5 treatment.

### E5 inhibits CXCL12- or MS-5- induced migration of acute myelocytic leukemia cells

Preclinical studies on different tumor models have revealed that CXCR4 activation mediates tumor cells migration towards CXCL12 expressing organs^26^,^27^. Bone marrow represents the most important organ for the homing of leukemia cells, in which stromal cells secret CXCL12 for recruiting those expressing CXCR4. The mechanisms of stroma-mediated protection for leukemia cells involve a complex interplay of stroma-produced cytokines, chemokines and adhesion molecules. Among the various mediators, CXCL12 and its cognate receptor CXCR4 are regarded as critical mediators of stromal/leukemic cell interactions. Therefore, in following chemotaxis studies, two methods were employed: (1) supplementing CXCL12 reagent directly in the culture medium in the bottom chamber of the transwell device; (2) mouse stromal cell MS-5 was seeded in the bottom chamber of the transwell device. These stromal cells can mimic the complex microenvironment of bone marrow as well as secrete CXCL12 constantly; the amount is comparable with that secreted by human bone marrow stromal cells^27^,^28^.

**Figure 3:**
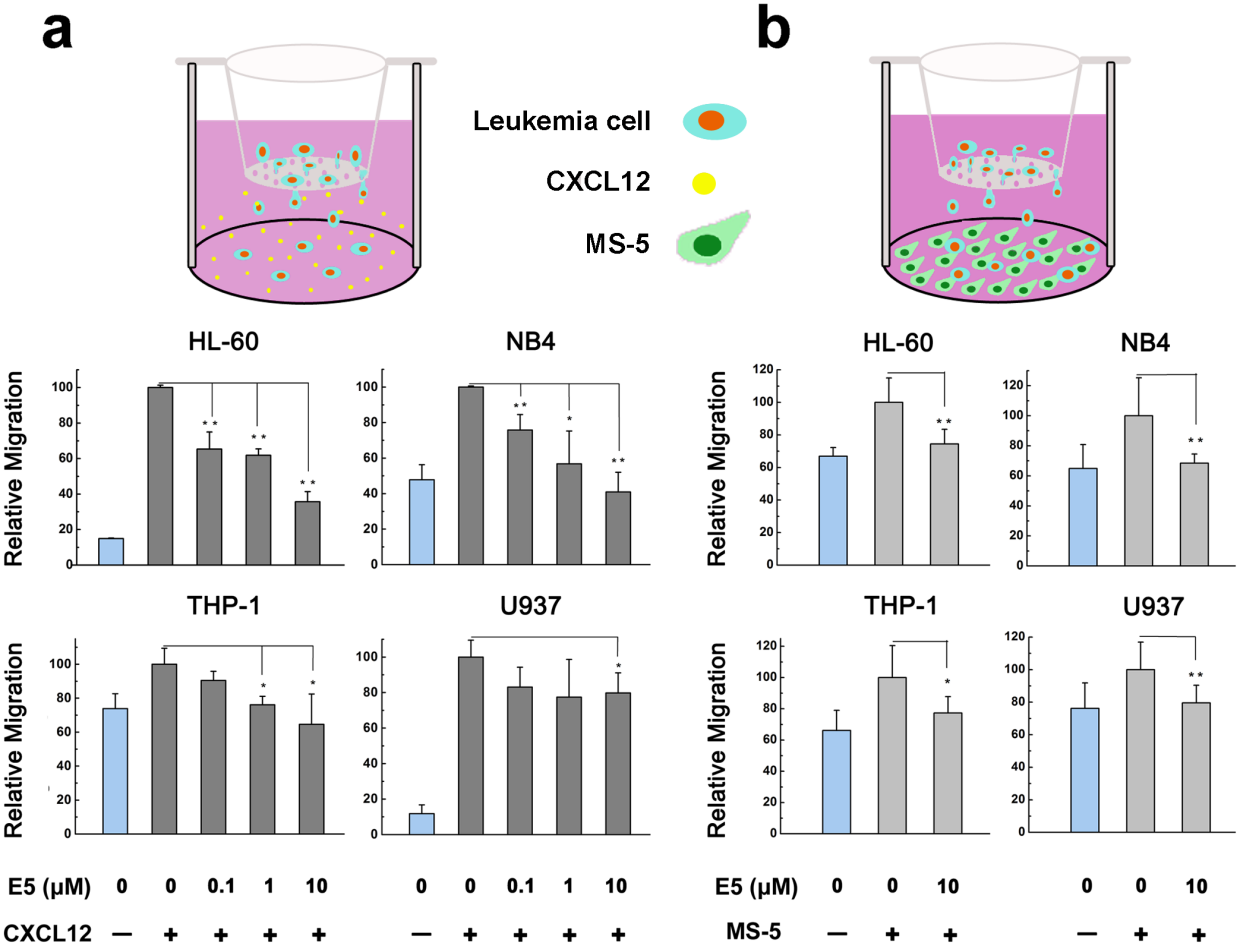
Inhibitory effect of E5 on the migration of multiple leukemia cell lines in the transwell assay. Leukemia cells were seeded in the upper well. CXCL12 was supplemented in the lower chamber (a) or secreted by MS-5 cells seeded in the lower chamber (b). Data are presented as mean ± SD (n = 3). The * represents significant difference from sample groups to the control group (*: *p* < 0.05, **: *p* < 0.01).

Leukemia cells were treated with E5 prior to be seeded in the upper chamber of the transwell device. As Figure 3a shows, in the absence of CXCL12, random migration of leukemia cells was in relative low levels. When CXCL12 was supplemented in the lower chamber of the transwell device, leukemia cells were enhanced to migrate into the chamber (set as 100% as control), and E5 significantly inhibited this effect in all cell lines tested in a concentration-dependent manner. In addition, the inhibitory effect of E5 is associated with the cell type. Treated with E5 at 0.1, 1.0 or 10 µM, the migration percentage was reduced to 65 ± 9.7%, 61 ± 3.6% or 36 ± 5.7% respectively for HL-60; 76 ± 8.8%, 57 ± 18.5% or 41 ± 11.1% for NB4; 90 ± 5.4%, 76 ± 5.0% or 65 ± 17.8% for THP-1; and 83 ± 11.2%, 77 ± 21.3% or 80 ± 11.3% for U937.

We further examined whether E5 treatment inhibits leukemia cells respond to MS-5-induced activation. In the MS-5 assay, the concentration of CXCL12 secreted by MS-5 cells was detected 8.4 ng/mL after 24 h of incubation. Similarly to CXCL12, MS-5 induced the migration of AML cells (set as 100% as control), and these effects were significantly inhibited by 10 µM of E5. The migration rate was reduced to 74 ± 9.0% for HL-60, 68 ± 6.1% for NB4, 77 ± 10.5% for THP-1, and 79 ± 10.9% for U937 (Fig. 3b). The inhibitory effect of E5 on MS-5 induced migration was also associated with cell types. These results suggest that E5 effectively inhibits both CXCL12-induced and MS-5-induced migration of the four acute myelocytic cell lines and more strongly inhibits the migration of HL-60 and NB4 either in the CXCL12 supplementing assay or in the MS-5 stromal cell assay.

### E5 inhibits leukemia cells from adhering to MS-5 cell layer

**Figure 4:**
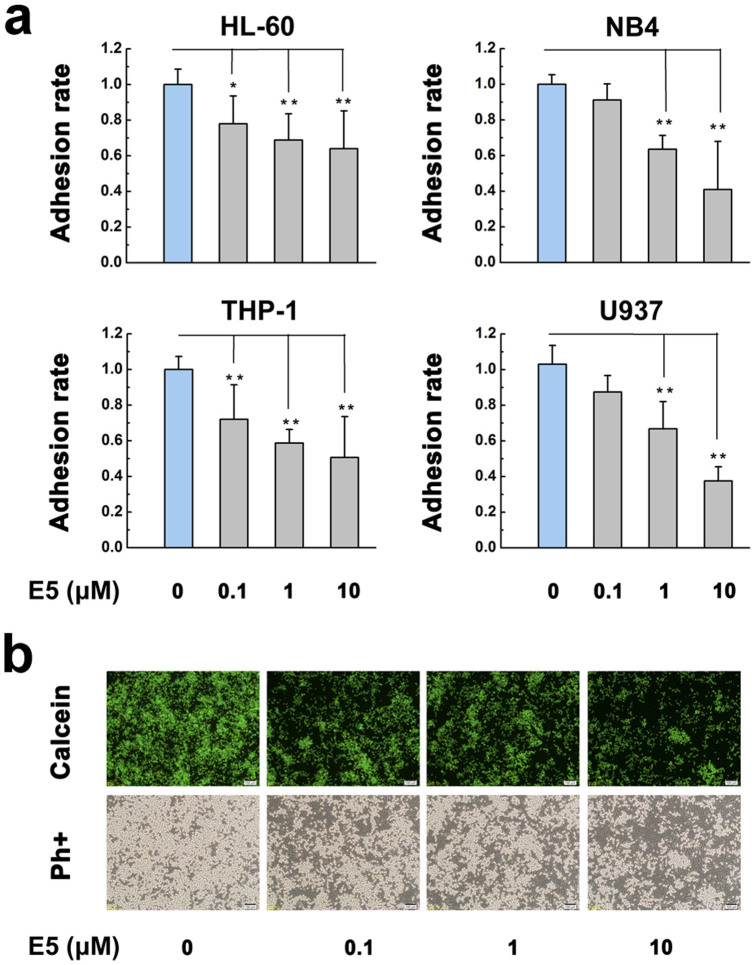
The inhibitory effect of E5 on the MS-5-mediated adhesion of multiple leukemia cell lines. MS-5 cells were seeded in the culture well to form a stromal cell layer. (a) The percentage of adhering leukemia cells on the MS-5 layer. The leukemia cells were pretreated with E5 at different concentrations. (b) Typical images of adhering NB4 cells pretreated with E5 at different concentrations. Leukemia cells were stained with calcein-AM (green). Data are presented as mean ± SD (n = 3). The * represents significant difference from sample groups to the control group (*: *p* < 0.05, **: *p* < 0.01).

It is well known that CXCL12 secreted by non-malignant stromal cells recruits leukemia cells to bone marrow. The recruited cells subsequently adhere to marrow stromal cells^29^,^30^,^31^. Direct cell interactions with bone marrow microenvironment components can provide survival, anti-apoptosis and drug resistance signals to the leukemia cells^13^,^32^. Hence we next investigated whether E5 interferes with the adhesive interaction between MS-5 and leukemia cells. Results clearly show that E5 significantly reduced the number of cells adhering to the MS-5 cell layer in a concentration dependent way in the four cell lines. The strongest inhibitory effect of E5 on the cell adhesion was reached to 36%, 59%, 49% and 63% for HL-60, NB4, THP-1 and U937 respectively. In the comparison, E5 more strongly inhibited the adhesion of NB4 and U937 among the four cell lines at 10 µM (Fig. 4).

### E5 affects CXCL12-induced cytoskeleton actin polymerization of leukemia cells

**Figure 5:**
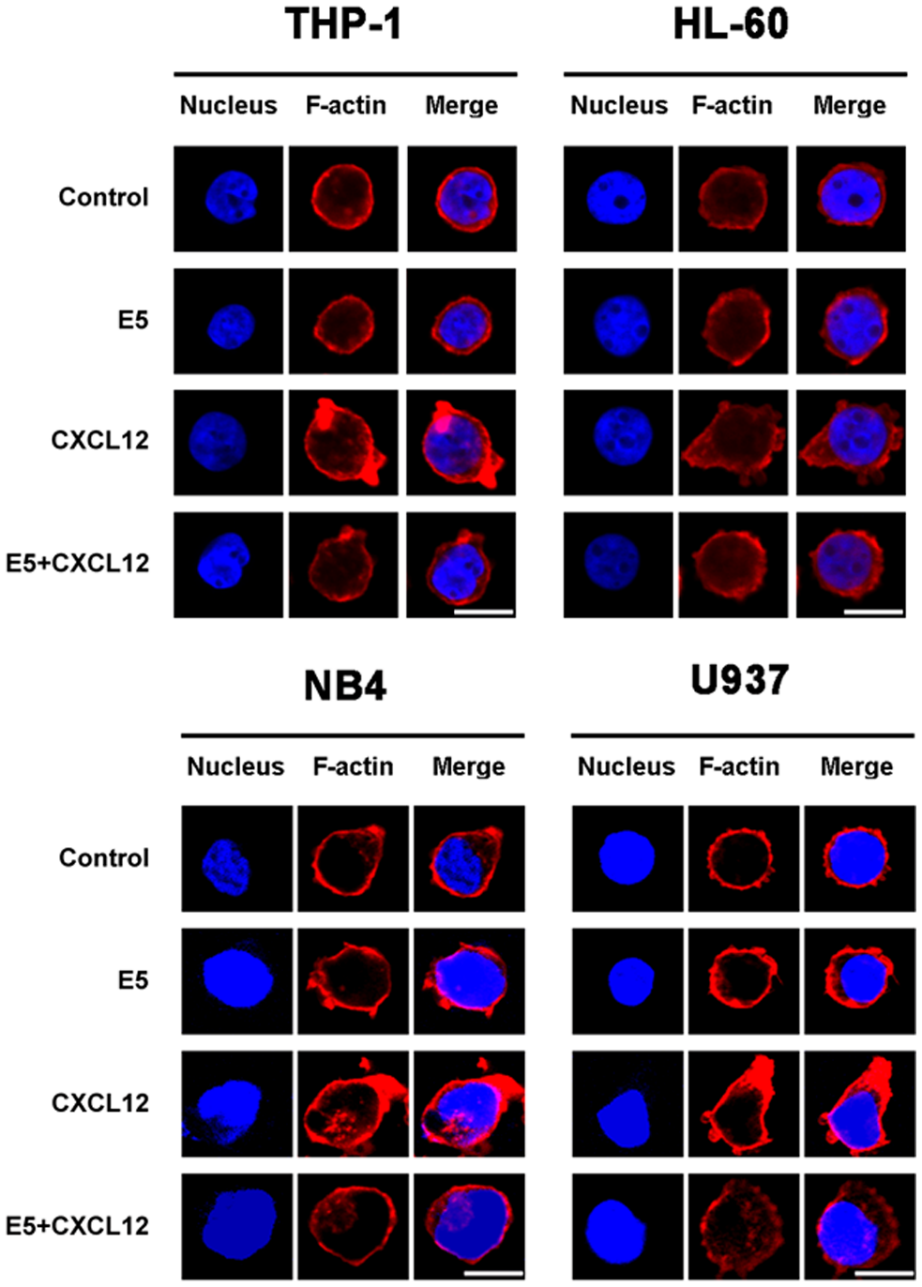
Stress fiber formation and reorganization of F-actin induced by CXCL12 upon E5 treatment. The control cells (first line) were treated with E5 alone (second line), CXCL12 alone (third line), or pretreated with E5 and then exposed to CXCL12 (bottom line). The F-actin was detected using TRITC-conjugated phalloidin (red fluorescence) and the nucleus was revealed with DAPI (blue fluorescence) by confocal microscopy. Scale bar represents 10 µm.

Both cell migration and adhesion are closely associated with the regulation of cytoskeleton actin microfilament^33^. To investigate whether E5 affects the cytoskeleton reorganization of leukemia cells, confocal assays was applied using a probe for F-actin. Results of the four leukemia cell lines were displayed representatively in Figure 5. In control cells F-actin dispersed throughout the whole cells uniformly. When incubated in the CXCL12-containing medium, the F-actin underwent edge ruffling, and uniform F-actin signals were mainly localized in the cells membrane, meanwhile condensed stress fiber formation was observed in both cells. These changes indicate cells were activated in response to the CXCL12. Treated with E5 at 10 µM prior to the CXCL12 exposure, F-actin signals diffused in the cytoplasm in nonhomogeneous status and decreased in the membrane. These results suggest that E5 inhibits the four cell lines from responding to the CXCL12 activation, F-actin signals stayed in the cytoplasm instead of ruffling to the cells edge, because CXCL12-induced stress fiber formation was attenuated and reorganization of F-actin was inhibited.

### E5 down-regulates CXCL12-induced Akt, Erk and p38 phosphorylation

**Figure 6:**
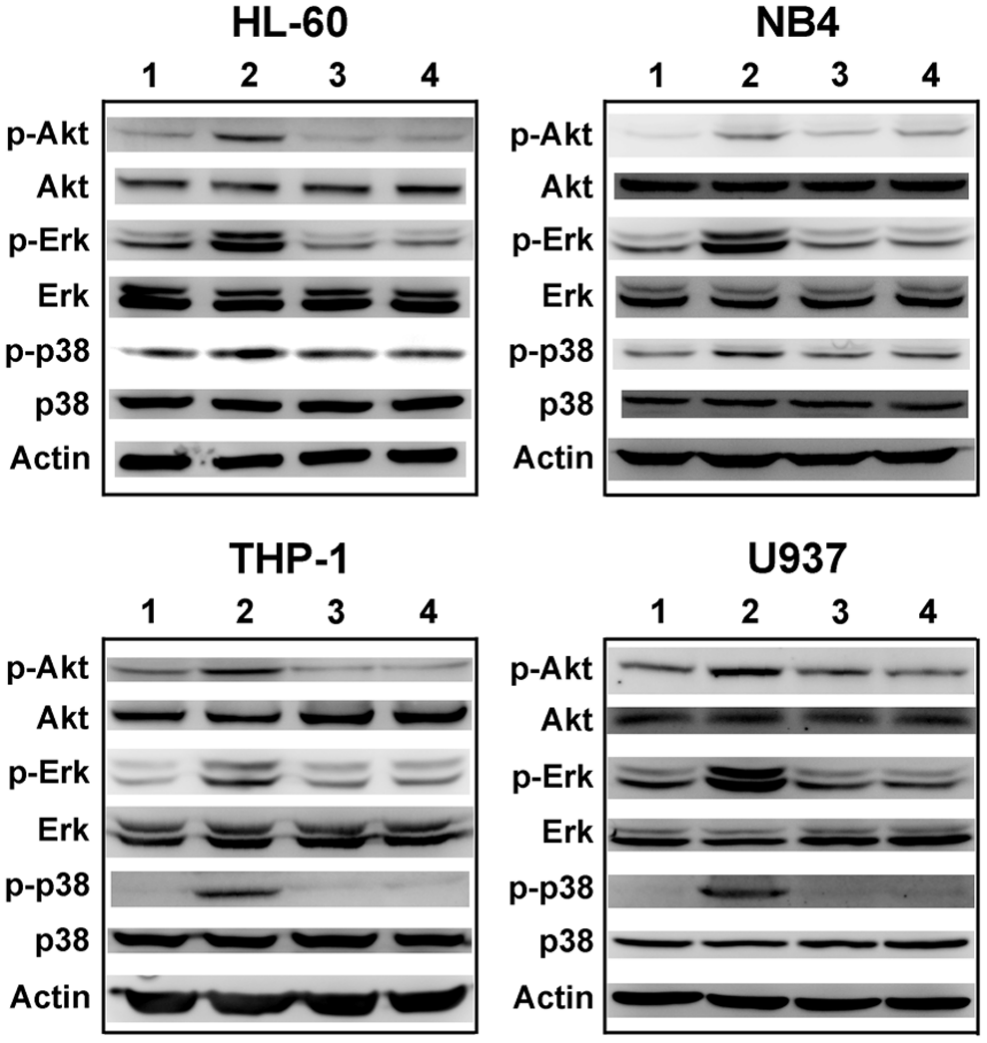
Effects of E5 on CXCL12-induced Erk, Akt and p38 activation of multiple leukemia cells. Lane 1: control, Lane 2: CXCL12 treatment for 10 minutes at 50 ng/mL (THP-1 and U937) or 200 ng/mL (HL-60 and NB4), Lane 3: treated with E5 at 10 µM for 1 h followed by the CXCL12 treatment, Lane 4: E5 treatment at 10 µM for 1 h. Full-length blots are presented in Supplementary Figures.

The CXCR4/CXCL12 interaction has been demonstrated to trigger Erk, Akt and p38/MAPK signaling, which accounts for the migration and adhesion of leukemia cells conferred by CXCL12^26^,^34^,^35^. To clarify whether E5 inhibits leukemia cells migration and adhesion to the stromal cells through affecting the intracellular signaling of CXCR4/CXCL12, we therefore conducted western blot analysis for activation of Erk (phospho-Erk), Akt (phosphor-Akt) and p38/MAPK (phospho-p38) in the four kinds of leukemia cell. The cells were treated with CXCL12 alone, E5 alone, or E5 prior to CXCL12 treatment. As shown in Figure 6, CXCL12 treatment significantly enhanced the phosphorylation levels of Erk, Akt and p38 (lane 2) in reference to that of control, while E5 not only down-regulated the basal phospho-Erk, phosphor-Akt and phospho-p38 levels (lane 4) but also abrogated the robust phosphorylation of Erk, Akt and p38 in the cells stimulated by CXCL12 (lane 3). These results clearly indicate that E5 inhibits AML cells from responding to CXCL12 stimulation by suppressing the expression of p-Erk, p-Akt and p-p38/MAPK.

### E5 prolongs the survival of NOD/SCID mice transplanted with human AML cells

**Figure 7:**
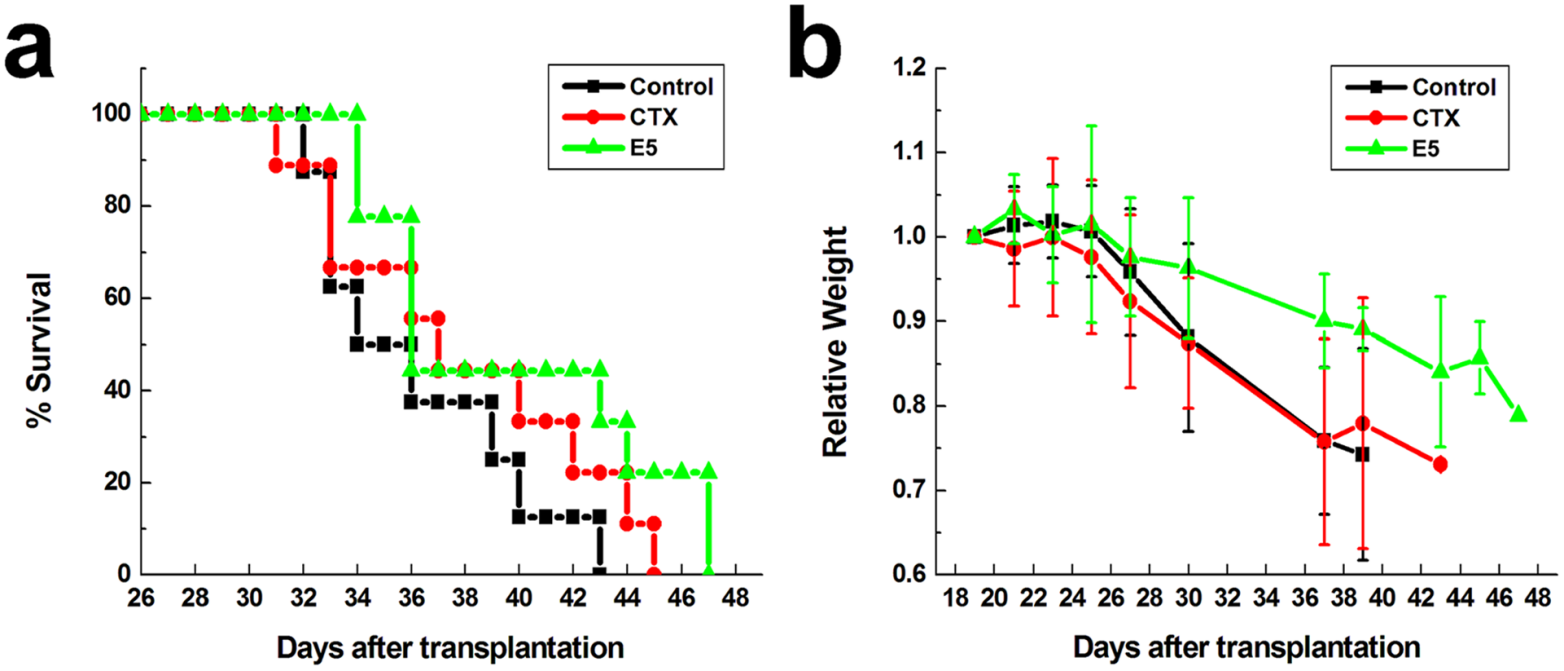
Therapeutic effects of E5 on human acute myelocytic leukemia in immunocompromised mice transplanted with HL-60 cells. NOD/SCID mice were intravenously injected with HL-60 cells (1 × 10^6^ cells per mouse). After 20 days, treatment was started with injection of sterile water, intraperitoneal injection of CTX (36 mg per kg) or subcutaneous injection of E5 (30 mg per kg) twice a week. (a) Survival of mice treated with sterile water, CTX or E5. (b) Relative weight of the mice during treatment.

Encouraged by the light of results *in vitro*, we translated our findings into an *in vivo* system. We injected HL-60 cells into sub-lethally irradiated NOD/SCID mice, allowed the cells to migrate to the bone marrow and to form an enlarged leukemia burden. Eighteen days after the injection, mice showed marked leukemic symptoms including paresis in the rear limbs, ruffled fur, and remarkably hunched posture in reference to mice of healthy control. On day 20 after the transplant, the leukemia mice were randomly divided into 3 groups: control (control, injected subcutaneously with sterile water), CTX (injected intraperitoneally with cyclophosphamide), and E5 (injected subcutaneously with E5). E5 or CTX was administrated to mice twice a week. As shown in Figure 7a, a prolonged survival was observed in those mice that received E5 treatment compared with the survival of CTX group and control group. Additionally, mice received E5 treatment had less reduction in the body weight (Fig. 7b) than those mice that received CTX treatment or in the control group. On day 36 after the transplant, the percentage of HL-60 cells in the leukocytes collected from spleen and bone marrow was analyzed with flow cytometry, which was 57.3% and 92.7% in the spleen and bone marrow respectively, detected by human CD33 expression, confirming the leukemia burden was formed and proving the effect of E5 in anti-acute myelocytic leukemia as well.

## Discussion

The migration and adhesion towards to the bone marrow stroma through CXCR4/CXCL12 axis are essential for the survival, proliferation and chemotherapy resistance of leukemia cells^2^,^3^,^4^, because bone marrow stroma provide a protective microenvironment and pro-survival signals for the homing leukemia cells. It has been widely recognized that CXCR4/CXCL12 axis plays an important role in leukemia development and relapse, and that provides an efficient target for leukemia therapy^6^,^9^. Among current developed CXCR4 antagonists, peptides are one attractive class to inhibit leukemia cells migration, invasion and adhesion, and to interfere with stromal cell-mediated tumor cell drug-resistance^14^.

Hematological malignancy includes various types; however, each kind of peptide antagonists is only studied in certain types of leukemia. For instances, the peptide T140 and its analogs TC14012 and TN14003 are reported with effective inhibitory roles mainly in lymphocytic leukemia cells, such as pre-B ALL cell line NALM6 and primary cells from pre-B ALL patients^16^,^17^ or B-CLL cells from patients, by down-regulating CXCL12-induced phosphorylation of Erk/MAPK and STAT3 signaling pathway^18^. RCP168 is another kind of CXCR4 peptide antagonist that inhibits CXCL12- or MS-5-induced migration of Jurkat or primary CLL cells from patients through down-regulating phosphorylation of Akt and Erk^23^. It is notable that up to date, peptide antagonists for acute myelocytic leukemia cells have been rarely published, though studies indicated that CXCR4 expression is associated with poor prognosis in patients with diploid AML^36^,^37^,^38^. Therefore, it is necessary and of significance to develop peptide antagonists targeting CXCR4 for uses in anti-AML, so that more therapeutic options can be provided to the patients.

The aim of this study was to explore the inhibitory effect of one novel peptide targeting CXCR4 on the activity of acute leukemia myelocytic cells. One of distinguished advantages of E5 over other peptide antagonists reported here is that it not only demonstrates significant inhibitory effects on the cell migration and adhesion in acute multiple myelocytic leukemia cell lines but exhibits anti-acute myelocytic leukemia efficacy in vitro and in vivo. The i*n vivo* study gives primary evidence that E5 can selectively kill leukemia cells directly and extends the lifespan of leukemia mice model and improve the living condition. Another attractive feature of E5 is that it neither induces nonmalignant cell apoptosis at a high concentration of 80 µM nor activates CXCR4 signal pathway of the leukemia cells. On the contrary, AMD3100 (a small chemical molecule blocking CXCR4/CXCL12 interaction) is reported a weak partial agonist (CXCL12-like) activity^39^,^40^. Therefore, E5 exhibits its therapeutic efficacy and safety in clinical application, which implies that E5 is applicable in treatments for more types of leukemia.

It is also noticeable that E5 not only inhibits leukemia cells from homing to the places where CXCL12 is secreted, but also mobilizes leukemia cells from a pre-established stromal feeder layer by disrupting the adhesion between the leukemia cells and the stromal cells, indicating it is able to drive the cells away from the protective microenvironment. This effect has its clinical implications in inhibiting the tumor cell metastasis and eradicating residual leukemia cells at locations where they would otherwise be protected by stroma. It is possible to use E5 to disrupt the interaction of leukemia cells with the stromal microenvironment, so as to destroy the growth and pro-survival signals provided by stromal cells. Moreover, CXCR4 is also highly expressed by many other malignant cell types^8^, such as breast cancer, lung cancer, melanoma, prostate cancer, and ovarian cancer, and closely associated with the metastasis, invasion and chemotherapy resistance of these solid tumors. Hence, broader uses of E5 can also be expected in those cancer therapies in combination with chemotherapies.

It can be noticed that most of currently reported peptide antagonists are derived from cells by bioengineering technology. As mentioned before, the peptide T140 and its analogs TC14012 and TN14003 are derived from naturally occurring peptides in horseshoe crabs. RCP168 is derived from viral macrophage inflammatory protein II. Contrary to those peptides, E5 is designed according to the sequence features of human CXCR4 and chemically synthesized, not derived from any natural compounds, which makes it more easily, flexibly and economically to be produced.

Taken together, our findings revealed the effect of E5 in inhibiting acute myeloid leukemia cells from responding to CXCL12- or MS-5 induced activation and eradicating leukemia cells in vitro and in vivo, which demonstrate the promising potentiality of E5 in leukemia therapies.

## Methods

### Peptide E5

In the current study, we tested a collection of chemically synthesized peptides based on the amyloid proteins and fragments of various proteins. Cell-based selection process is performed for screening of the peptide ligands with high binding affinity. This collection of peptides were examined in our previous studies for unraveling various interaction, hydrogen bond, electrostatic and hydrophobic interactions between side chains, in the peptide assemblies using scanning probe microscopy^41^,^42^,^43^. A peptide E5 (GGRSFFLLRRIQGCRFRNTVDD) was identified by the cell-based selection. Peptides used in this study were purchased from GL Biochem (Shanghai) Ltd.

### Cell culture

The acute promyelocytic leukemia cell HL-60 and NB4, acute monocytic leukemia cell THP-1, myelomonocytic leukemia cell U937 and human umbilical vein cell ea.hy926 were purchased from the Cell Resource Center of Chinese Academy of Medical Sciences (Beijing, China). Murine stromal cell line MS-5 was kindly provided by Professor Bin Yin, the Cyrus Tang Hematology Center, Soochow University, China. Those cells were cultured in 5% CO_2_ at 37°C in RPMI 1640 (Hyclone Thermo Scientific) supplemented with phenol red, 10% fetal bovine serum (FBS; Hyclone Thermo Scientific), 100 U/mL penicillin, 100 U/mL streptomycin.

### The CXCR4 level of human leukemia cells

Leukemia cells (HL-60, NB4, THP-1 and U937) of 5 × 10^5^ were incubated with mouse monoclonal anti-human CXCR4 antibody (12G5; R&D Systems, Minneapolis, MN) in BSA/PBS for 1 h at 4°C and then treated with goat anti-mouse-IgG-FITC (BioLegend, San Diego, CA). Resulting cells were subjected to C6 Accuri® flow cytometer (Accuri Cytometers, Ann Arbor, MI). The 1.5 × 10^4^ cells were collected, acquired data were analyzed by CFlow Plus software.

### Affinity assay

Leukemia cells of 5 × 10^5^/well (HL-60, NB4, THP-1 and U937) were seeded in 24-well plates in 500 µL serum-free medium (opti-MEM; Life Technologies, Grand Island, NY). The stock solution of biotin-E5 was dissolved in the sterile water. In the time-effect assay, cells were incubated with biotin-E5 at 10 µM (37°C, 5% CO_2_) and collected at indicated time points (0–4 h). In the concentration-effect assay, cells were incubated with biotin-E5 at different concentrations (0–60 µM) for 2 h. Cells were then washed with BSA/PBS and incubated with FITC-conjugated Streptavidin (BioLegend, San Diego, CA) at 4°C. After washed with BSA/PBS, the cells were subjected to C6 Accuri® flow cytometer. The 1.5 × 10^4^ cells were collected, acquired data were analyzed by CFlow Plus software.

### Cytotoxicity evaluation

FITC-Annexin V/PI (eBioscience, Vienna, Austria) double staining was used to determine the apoptosis of leukemia cells (**HL-60, NB4, THP-1 and U937**), MS-5 and ea.hy926 upon E5 according to the manufacturer's protocol. Cells were seeded in 24-well plates in which E5 was added at different concentrations for incubation of 24 h. Collected and washed with PBS, cells were resuspended in the binding buffer (10 mM Hepes/NaOH, pH 7.4, 140 mM NaCl, 2.5 mM CaCl_2_) and FITC-Annexin V and PI were added followed by 10 min incubation at room temperature and subjected to C6 Accuri® flow cytometer. The 1.5 × 10^4^ cells were measured, acquired data were analyzed by CFlow Plus software.

### Western blot analysis for caspase-3 activation

Leukemia cells (HL-60, NB4, THP-1 and U937) were seeded in 24-well plates at 5 × 10^5^ cells/well in which E5 was added at different concentrations for incubation of 24 h. Collected and washed with PBS, cells were lysed in RIPA lysis buffer (Beyotime Biotechnology, Haimen, China) supplemented with protease inhibitors (Sigma-Aldrich, USA) on the ice for 30 min. The lysates were clarified by centrifugation at 15000 rpm for 10 min. Equal protein (30 µg) was loaded and separated on 12% polyacrylamide gels (Applygen, Beijing, China), and transferred to PVDF membranes (0.2 µm; Millipore, Bedford, MA). Cleaved caspase-3 and β-Actin were probed with specific primary antibodies (Cell Signaling Technology, Beverly, MA) and secondary antibodies conjugated to HRP (Zhongshan Goldenbridge biotechnology Co, Beijing, China). The immunocomplex on the membrane was visualized using Image Quant LAS 4000 (GE Healthcare) with Immobilon ® Western HRP Substrate luminol reagent and peroxide solution (Millipore, Billerica, MA).

### CXCL12 secretion by MS-5

The murine stromal cell line MS-5 cells of 1 × 10^5^/well were seeded in 24-well plate with 800 µL medium and incubated for 24 h at 37°C. Then the medium was collected and measured using Mouse CXCL12 Quantikine Elisa Kit (R&D Systems, Minneapolis, MN) according to the manufacturer's instruction. The concentration of CXCL12 secreted by MS-5 cells was determined by the absorbance at 450 nm/570 nm using a BioTek Synergy^TM^ 4 Hybrid Multi-Mode Microplate Reader (BioTek Instruments, Winooski, VT). The fresh medium was set as control.

### Cell migration assay

The transwell assay was applied using millicell hanging cell culture inserts (5 µm for THP-1 and U937, 8 µm for HL-60 and NB4; Millipore, Switzerland). Leukemia cells of HL-60, NB4, THP-1, and U937 were treated with E5 at different concentrations in serum-free medium opti-MEM at 37°C for 1 h and then seeded into the upper chambers of inserts (2 × 10^5^/chamber). The complete RPMI 1640 medium of 800 µL with CXCL12 (R&D Systems, Minneapolis, MN) at 50 ng/mL for THP-1 and U937 or 200 ng/mL for HL-60 and NB4 was added in the lower chamber. After 24 h incubation, cells migrating to lower chambers were counted using TC10^TM^ automated cell counter (Bio-RAD, Hercules, CA).

To examine the migration mediated by stromal cells, MS-5 cells of 1.5 × 10^5^/well were seeded and incubated overnight in the lower chamber and treated with 10 µg/mL Mitomycin-C (Kyowa Hakko Kogyo, Tokyo, Japan) for 2 h. Next, MS-5 cells were washed with PBS and incubated in 800 µL of fresh culture medium for 20 h. Leukemia cells seeded in the upper chambers were fluorescently labeled with 1 µg/mL of Calcein-AM (Molecular Probes, Eugene, OR) and then treated with E5 at 10 µM for 1 h prior to be hanged on the lower chambers. After 6 h incubation in the dark, cells migrating to the lower chambers were collected and the fluorescence was measured using the BioTek Synergy^TM^ 4 Hybrid Multi-Mode Microplate Reader at an excitation wavelength of 488 nm and an emission wavelength of 514 nm.

### Adhesion Assays

MS-5 cells of 1.5 × 10^5^/well were seeded in the 24-well plate and incubated overnight to form a stromal cell layer. Leukemia cells of HL-60, NB4, THP-1 and U937 were fluorescently labeled with 1 µg/mL Calcein-AM and treated with E5 for 2 h at different concentrations in the serum-free medium opti-MEM. After that, 5 × 10^5^ of fluorescent leukemia cells were added to each of the wells with MS-5 cell layers, followed by 1 h incubation at 37°C. Non-adherent cells were gently collected and measured by Synergy^TM^ 4 Hybrid Multi-Mode Microplate Reader at an excitation wavelength of 488 nm and an emission wavelength of 514 nm, and adherent cells were imaged by the fluorescent microscope (Olympus, Tokyo, Japan).

### Confocal microscopy analyses of actin microfilament

The four cell lines including HL-60, U937, NB4 and THP-1 were seeded in 24-well plates at 5 × 10^5^ cells/well in serum-free medium supplemented with E5 at 10 µM and incubated for 1 h. Next, CXCL12 was added into the medium at 200 ng/mL for HL-60 and NB4 or at 50 ng/mL for U937 and THP-1, followed by incubation of 1 h at 37°C. The culture medium without CXCL12 was set as control. The cells were then fixed in paraformaldehyde/PBS for 30 min. After rinsing with PBS, cells were permeabilized in 0.5% Triton X-100/PBS for 5 min at 4°C and then washed with PBS. After that, cells were incubated in BSA/PBS for 5 min at 37°C and subsequently labeled with 2 µg/mL of tetramethylrhodamine-conjugated phalloidin (Sigma-Aldrich, USA) for actin staining for 40 min and washed with 0.5% Tween 20/PBS. Resulting cells were mounted with an aqueous mounting medium containing DAPI (Zhongshan Goldenbridge biotechnology Co, Beijing, China). Images were taken from a laser confocal microscope (UltraVIEW VoX; Perkin Elmer, Waltham, MA) and analyzed by Volocity-5 software (Perkin Elmer).

### Western blot analysis for phosphorylation of Erk, Akt and p38

Leukemia cells (HL-60, NB4, THP-1 and U937) were maintained overnight in RPMI 1640 that contained 0.5% FBS at 5 × 10^5^ cells/well in 24-well plate, followed by treated with E5 at 10 µM for 1 h. After that, CXCL12 (200 ng/mL for HL-60 and NB4 or 50 ng/mL for U937 and THP-1) was added into the medium for 10 minutes at 37°C. Then adding excess cold PBS, cells were collected and lysed in RIPA lysis buffer supplemented with protease inhibitors and phosphatase inhibitors mixture (Applygen, Beijing, China) on the ice for 30 min. The lysates were clarified by centrifugation at 15000 rpm for 10 min. Equal protein (30 µg) was loaded and separated on 12% polyacrylamide gels, and transferred to PVDF membranes (0.45 µm; Millipore, Bedford, MA). Phosphorylated and total proteins were probed with specific primary antibodies (Erk, phosphorylated-Erk, Akt, phosphorylated-Akt, p38, phosphorylated-p38, β-Actin; Cell Signaling Technology, Beverly, MA) and secondary antibodies conjugated to HRP. The immunocomplex on the membrane was visualized using Image Quant LAS 4000 with Immobilon ® Western HRP Substrate luminol reagent and peroxide solution.

### Treatment in leukemia mice model

Five-week old female NOD/SCID mice were maintained in the Experimental Animal Center at the Institute of Basic Medical Sciences, Chinese Academy of Medical Sciences (Beijing, China) under specific pathogen-free conditions. All the animal experiments reported herein were carried out in accordance with the approved guideline and approved by the committee on the Animal Care and Use of Institute of Basic Medical Sciences, Chinese Academy of Medical Sciences & Peking Union Medical College. Animals were acclimatized to laboratory conditions for 1 week prior to experiments. HL-60 cells (1 × 10^6^) suspended in 100 µL EDTA/PBS were injected intravenously into the sublethally irradiated (250 cGy) NOD/SCID mice. At about 20 days after transplantation, mice showed leukemia signs. Then the mice were treated with E5 (30 mg/kg, n = 9) cyclophosphamide (CTX, 36 mg/kg, n = 9) or sterile water (n = 8) twice a week. E5 or sterile water was injected subcutaneously and CTX was injected intraperitoneally. Mice were monitored for the condition and weight loss. Another two mice transplanted with HL-60 and without any other treatment were sacrificed on day 36 after transplantation. The spleen and bone marrow from femur flushing were collected to prepare single cell suspension and erythrocytes were excluded using the blood cells lysis buffer (Beckman Coulter, Krefeld, Germany). PE anti-human CD33 antibody (BioLegend, San Diego, CA) was used to identify HL-60 with flow cytometry.

### Statistical analysis

All experiments were carried out in triplicate and Student's t-test was performed to assess statistical significance of the results (*P < 0.05 and **P < 0.01).

## Author Contributions

C.W. and H.X. designed the peptide and experiments, made data analysis and wrote the main manuscript. X.L. conducted main experiments and made data analysis; prepared figures and discussed all sections of the manuscript with the corresponding authors. H.G. conducted some experiments and prepared the figures. Y.Y. designed the peptide and discussed part sections of the manuscript with the corresponding authors. J.M. and J.L. made data analysis and discussed part sections of the manuscript with the corresponding authors. All authors reviewed the manuscript.

## Supplementary Material

Supplementary Information supplementary information
