# Supplementary material for: A designed peptide targeting CXCR4 displays anti-acute myelocytic leukemia activity in vitro and in vivo
Source: Sci Rep. 2014 Oct 14;4:6610. doi: 10.1038/srep06610 (PMC4196105; doi:10.1038/srep06610)
Supplement: Supplementary Information [file srep06610-s1.doc]

**A designed peptide targeting CXCR4 displays anti-acute myelocytic leukemia activity in vitro and in vivo**

Xiaojin Li^1^, Hua Guo^1^, Yanlian Yang^2^, Jie Meng^1^, Jian Liu^1^, Chen Wang^2*^, Haiyan Xu^1*^

1. Institute of Basic Medical Sciences, Chinese Academy of Medical Sciences & Peking Union Medical College，Beijing 100005，P. R. China.
2. National Center for Nanoscience and Technology, Beijing 100190, P. R. China

^*^ Corresponding authors: Haiyan Xu: [xuhy@pumc.edu.cn](mailto:xuhy@pumc.edu.cn) and Chen Wang: [wangch@nanoctr.cn](mailto:wangch@nanoctr.cn)

**
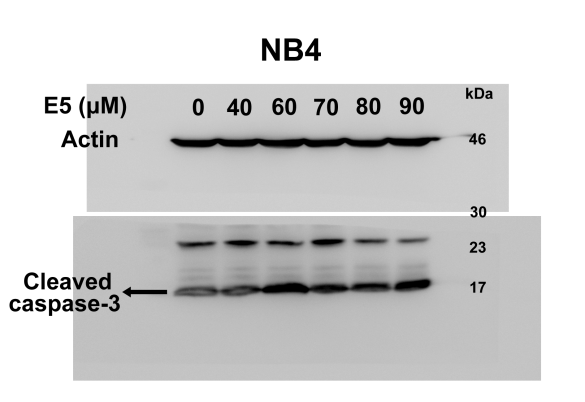

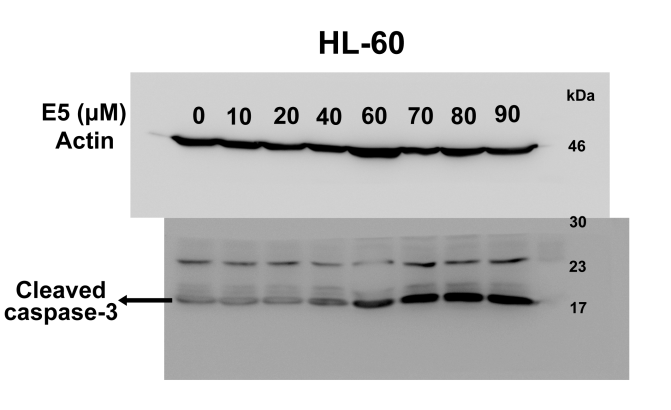
**

**
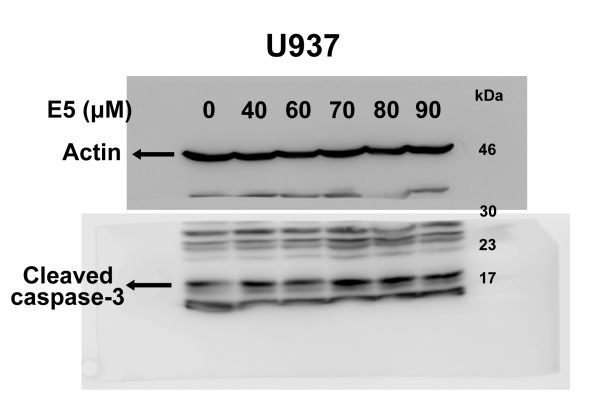

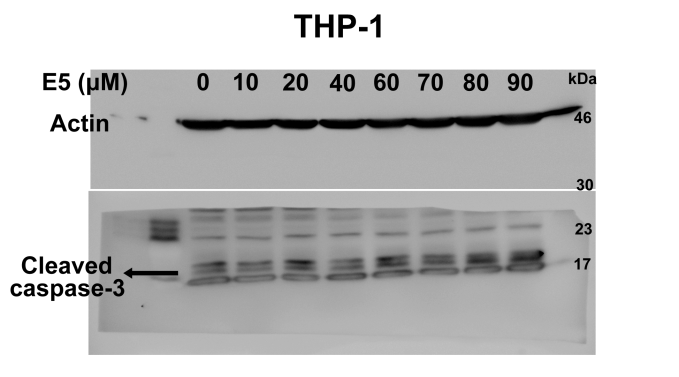
**

**Supplementary Figure 1**

**The cytotoxicity of E5 on different cells.** E5 induces the activation of caspase-3 signaling in concentration-dependent manner in HL-60, NB4, U937 and THP-1.


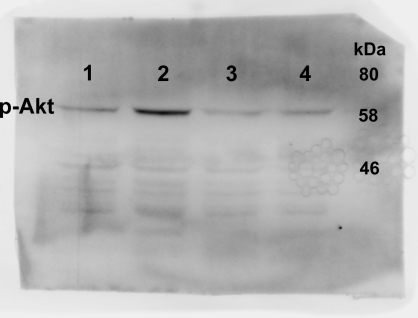

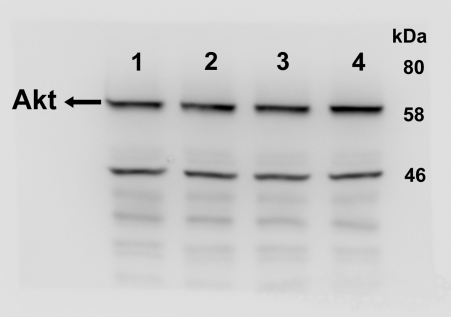


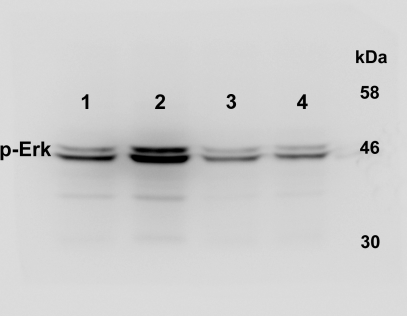

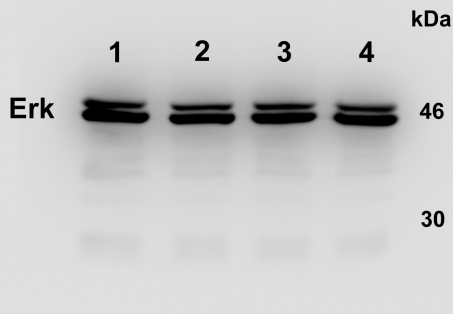


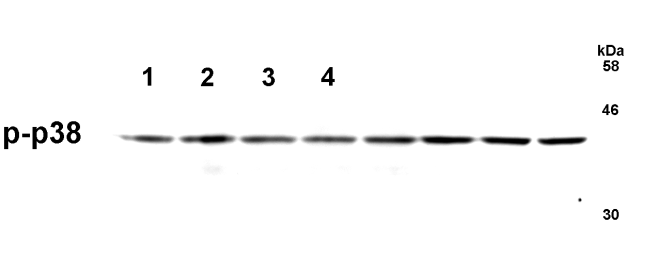

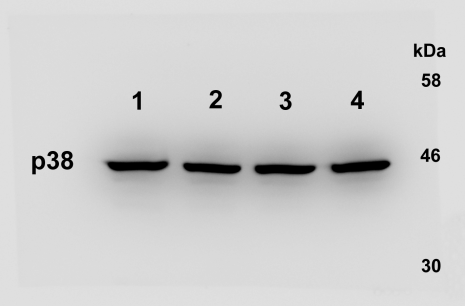


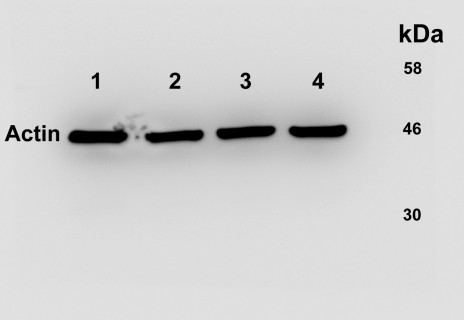


**Supplementary Figure 2**

**Effects of E5 on CXCL12-induced Akt, Erk and p38 activation of HL-60 cells.** Lane 1: control, Lane 2: CXCL12 treatment for 10 minutes, Lane 3: treated with E5 at 10 μM for 1 h followed by the CXCL12 treatment, Lane 4: E5 treatment at 10 μM for 1 h.


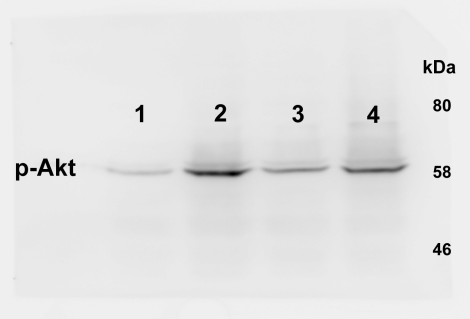

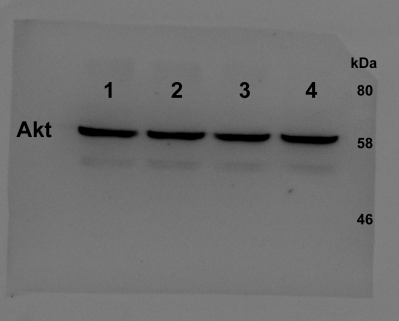


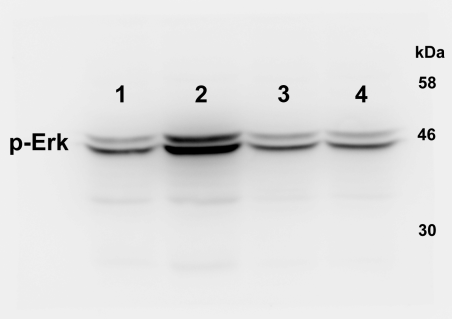

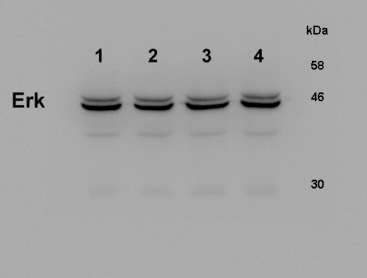


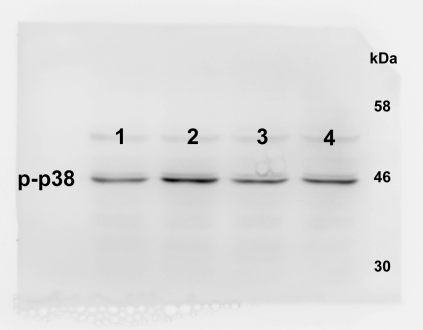

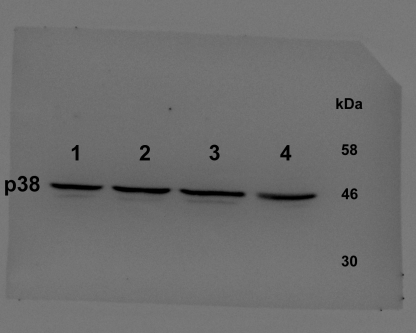


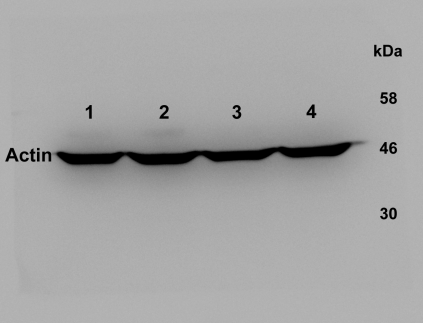


**Supplementary Figure 3**

**Effects of E5 on CXCL12-induced Akt, Erk and p38 activation of NB4 cells.** Lane 1: control, Lane 2: CXCL12 treatment for 10 minutes, Lane 3: treated with E5 at 10 μM for 1 h followed by the CXCL12 treatment, Lane 4: E5 treatment at 10 μM for 1 h.


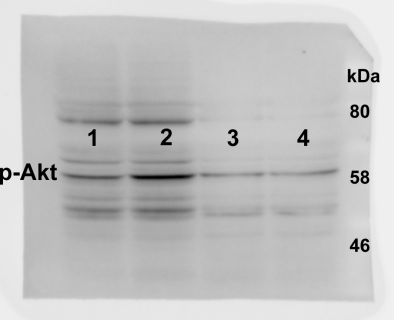

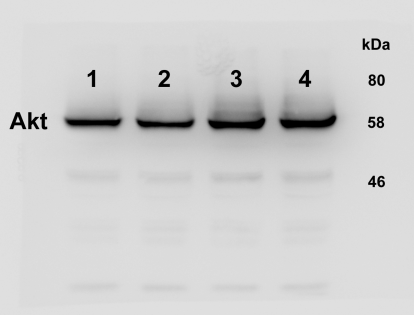


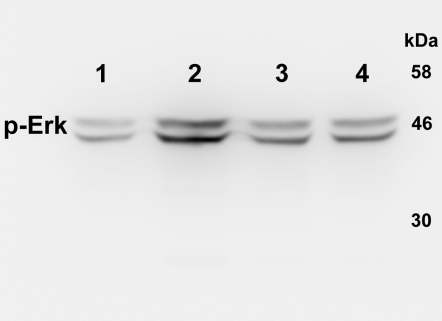

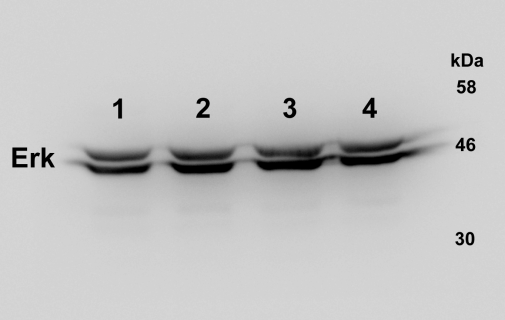


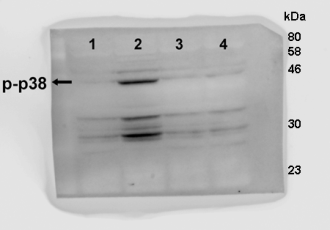

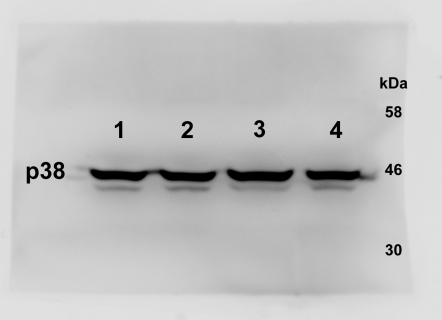


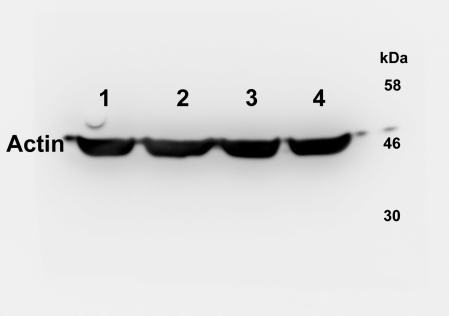


**Supplementary Figure 4**

**Effects of E5 on CXCL12-induced Akt, Erk and p38 activation of THP-1 cells.** Lane 1: control, Lane 2: CXCL12 treatment for 10 minutes, Lane 3: treated with E5 at 10 μM for 1 h followed by the CXCL12 treatment, Lane 4: E5 treatment at 10 μM for 1 h.


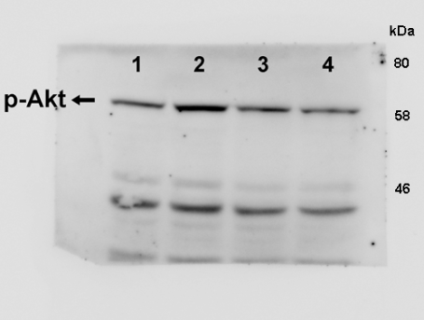

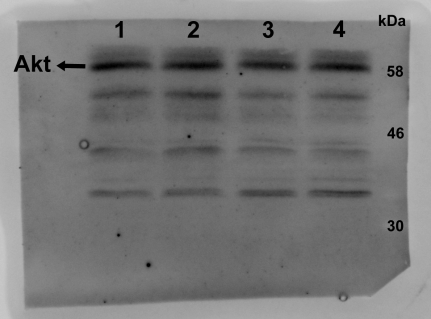


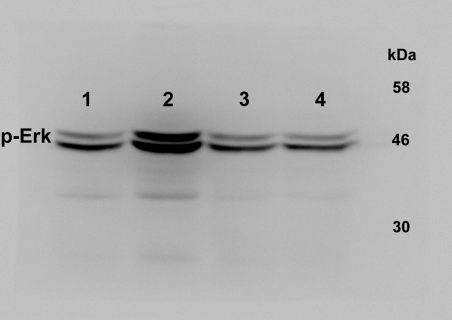

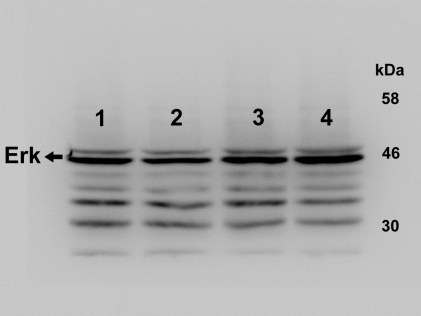


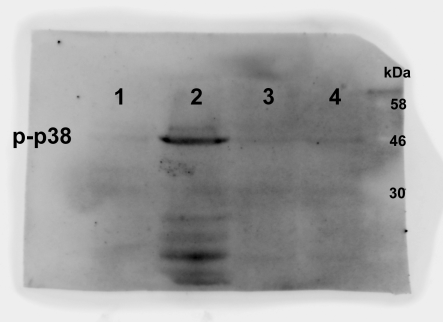

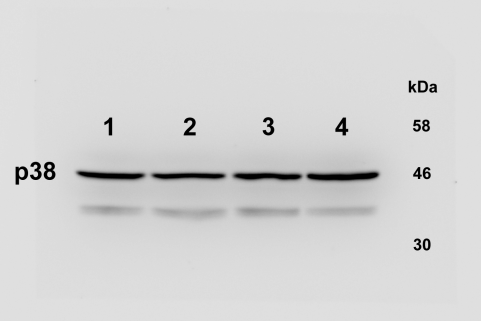


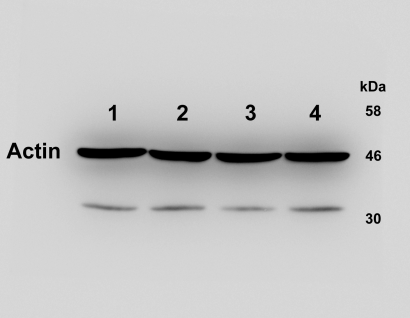


**Supplementary Figure 5**

**Effects of E5 on CXCL12-induced Akt, Erk and p38 activation of U937 cells.** Lane 1: control, Lane 2: CXCL12 treatment for 10 minutes, Lane 3: treated with E5 at 10 μM for 1 h followed by the CXCL12 treatment, Lane 4: E5 treatment at 10 μM for 1 h.
